# The healthy food environment policy index: findings of an expert panel in New Zealand

**DOI:** 10.2471/BLT.14.145540

**Published:** 2015-03-17

**Authors:** Stefanie Vandevijvere, Clare Dominick, Anandita Devi, Boyd Swinburn, for the International Network for Food and Obesity/non-communicable diseases Research, Monitoring and Action Support

**Affiliations:** a University of Auckland, School of Population Health, Private Bag 92019, Auckland Mail Centre, 1142 Auckland, New Zealand.

## Abstract

**Objective:**

To assess government actions to improve the healthiness of food environments in New Zealand, based on the healthy food environment policy index.

**Methods:**

A panel of 52 public health experts rated the extent of government implementation against international best practice for 42 indicators of food environment policy and infrastructure support. Their ratings were informed by documented evidence, validated by government officials and international benchmarks.

**Findings:**

There was a high level of implementation for some indicators: providing ingredient lists and nutrient declarations and regulating health claims on packaged foods; transparency in policy development; monitoring prevalence of noncommunicable diseases and monitoring risk factors for noncommunicable diseases. There was very little, if any implementation of the following indicators: restrictions on unhealthy food marketing to children; fiscal and food retail policies and protection of national food environments within trade agreements. Interrater reliability was 0.78 (95% confidence interval, CI: 0.76–0.79). Based on the implementation gaps, the experts recommended 34 actions, and prioritized seven of these.

**Conclusion:**

The healthy food environment policy index provides a useful set of indicators that can focus attention on where government action is needed. It is anticipated that this policy index will increase accountability of governments, stimulate government action and support civil society advocacy efforts.

## Résumé

**Objectif:**

Évaluer les actions gouvernementales visant à améliorer la salubrité des environnements alimentaires en Nouvelle-Zélande en fonction de l'index des politiques relatives à un environnement alimentaire sain.

**Méthodes:**

Un groupe de 52 experts en santé publique a évalué l'ampleur de la mise en œuvre gouvernementale par rapport aux meilleures pratiques internationales concernant 42 indicateurs de politiques relatives à l'environnement alimentaire et de soutien aux infrastructures. Leur évaluation reposait sur des informations documentées, validées par des représentants du gouvernement et des références internationales.

**Résultats:**

Certains indicateurs présentaient un haut niveau de mise en œuvre : communication des listes d'ingrédients et des informations nutritionnelles et réglementation des allégations relatives à la santé sur les aliments emballés ; transparence dans l'élaboration des politiques ; suivi de la prévalence des maladies non transmissibles et suivi des facteurs de risque pour les maladies non transmissibles. La mise en œuvre était faible, voire inexistante, en ce qui concerne les indicateurs suivants : restrictions relatives à la commercialisation de produits alimentaires malsains pour les enfants ; politiques budgétaires et de commerce de détail alimentaire et protection des environnements alimentaires nationaux dans les accords commerciaux. La fiabilité interévaluateurs était de 0,78 (intervalle de confiance, IC, à 95 % : 0,76 - 0,79). Compte tenu des lacunes dans la mise en œuvre, les experts ont recommandé 34 actions et donné un degré de priorité élevé à sept d'entre elles.

**Conclusion:**

L'index des politiques relatives à un environnement alimentaire sain donne accès à une série d'indicateurs utiles qui peuvent attirer l'attention sur les champs d'action restant à couvrir par le gouvernement. Cet index des politiques devrait augmenter la responsabilité des gouvernements, stimuler l'action gouvernementale et soutenir les efforts de sensibilisation de la société civile.

## Resumen

**Objetivo:**

Evaluar las acciones gubernamentales para mejorar la salubridad de los entornos alimentarios en Nueva Zelandia, sobre la base del índice de políticas para un entorno alimentario saludable.

**Métodos:**

Un grupo de 52 expertos en salud pública evaluó el alcance de la aplicación gubernamental con respecto a las mejores prácticas internacionales para 42 indicadores de políticas de entorno alimentario y apoyo infraestructural. Sus calificaciones se basaron en datos documentados, validados por funcionarios públicos y parámetros de referencia internacionales.

**Resultados:**

Se detectó un alto nivel de aplicación para algunos indicadores: provisión de listas de ingredientes y declaraciones nutricionales y regulación de las declaraciones sanitarias de los alimentos envasados; transparencia en el desarrollo de la política; monitoreo de la prevalencia de enfermedades no transmisibles y de sus factores de riesgo. Hubo muy poca implementación, o ninguna, de los siguientes indicadores: restricciones en la publicidad de alimentos insalubres para los niños; políticas fiscales y de venta de alimentos al por menor y protección de entornos alimentarios nacionales dentro de los acuerdos comerciales. La fiabilidad de los evaluadores fue del 0,78 (intervalo de confianza, IC, del 95%: 0,76–0,79). Basándose en las brechas de aplicación, los expertos recomendaron 34 acciones, de las cuales priorizaron siete.

**Conclusión:**

El índice de políticas para un entorno alimentario saludable proporciona un conjunto útil de indicadores que pueden centrar la atención en aquellos lugares en los cuales se necesita acción gubernamental. Se prevé que este índice de políticas aumentará la rendición de cuentas de los gobiernos, estimulará la acción gubernamental y apoyará las actividades de promoción de la sociedad civil.

## ملخص

**الغرض:**

تقييم إجراءات الحكومة لتحسين مدى سلامة البيئات الغذائية في نيوزيلندا، استناداً إلى مؤشر سياسة البيئة الغذائية الصحية.

**الطريقة:**

قام فريق مكون من 25 خبيراً في مجال الصحة العمومية بتقييم مدى التزام الحكومة بتنفيذ الممارسة العالمية المثلى لعدد 42 مؤشراً لسياسة البيئة الغذائية ودعم البنية التحتية. ارتكزت تقييماتهم على أدلَّة موثقة، وتم توثيق مصدوقيتها بواسطة مسؤولين حكوميين ومعايير عالمية. ****

**النتائج:**

كان هناك تنفيذ بمستوى مرتفع لبعض المؤشرات: توفير قوائم المكونات وبيانات المغذيات واللوائح الصحية التنظيمية على أغلفة المنتجات الغذائية المعبأة؛ والشفافية في تطوير السياسات؛ ومراقبة انتشار الأمراض غير السارية؛ ومراقبة عوامل الخطر للأمراض غير السارية. كان هناك تنفيذ محدود للغاية، إن وُجد، للمؤشرات التالية: القيود المفروضة على تسويق الغذاء غير الصحي للأطفال؛ والسياسات المالية وسياسات تجارة المنتجات الغذائية بالتجزئة؛ وحماية البيئات الغذائية الوطنية في إطار الاتفاقيات التجارية. كانت المعولية بين القائمين بالتقييم 0.78 (بنسبة أرجحية مقدارها 95%: فاصل الثقة: 0.76–0.79) بالاستناد إلى ثغرات التنفيذ، أوصى الخبراء باتخاذ 34 إجراءً ومنحوا الأولوية لسبعة منها.

**الاستنتاج:**

يوفر مؤشر سياسة البيئة الغذائية الصحية مجموعة مفيدة من المؤشرات التي يمكن أن تركز الانتباه على ما يستدعي اتخاذ إجراء حكومي. من المتوقع أن مؤشر السياسة هذا سيزيد من مساءلة الحكومات، ويحفز الإجراءات الحكومية، ويدعم جهود المناصرة من المجتمع المدني.

## 摘要

**目的:**

旨在根据卫生食品环境政策衡量指标，评价政府为改善新西兰食品环境的卫生程度而采取的行动。

**方法:**

由 52 个公共卫生专家组成的小组针对食品环境政策和基础设施支持的 42 个指标，参比国际最佳实践对政府实施的程度进行了评级。他们提供的评级结果均有记录在册的证据，并由政府官员按照国际基准进行验证。

**结果:**

实施程度高的一些指标包括：在包装食品上提供配料表和营养成分表以及规范的健康声明；政策制定的透明度；监控非传染性疾病的患病率以及监控非传染性疾病的风险因素。实施程度很低的一些指标如下：对于对儿童不健康食品市场营销的限制；财政和食品零售政策以及在贸易协议中对国家食品环境的保护。评级者间信度为 0.78（95% 置信区间：CI: 0.76–0.79）。基于实施差距，专家建议采取 34 项行动，其中 7 项为优先行动。

**结论:**

卫生食品环境政策衡量指标提供了一系列有用的指标，可以让政府集中精力专注于需要采取行动的方面。我们预计该政策衡量指标将增强政府的责任感，激励政府采取行动并支持民间社会的倡导工作。

## Резюме

**Цель:**

Оценить действия правительства по улучшению условий для здорового питания в Новой Зеландии на основе индекса эффективности политики здорового питания.

**Методы:**

Комиссия из 52 экспертов в области общественного здравоохранения провела оценку реализации политики здорового питания и ее инфраструктурной поддержки правительством на основании 42 показателей согласно передовой международной практике. Результаты оценки основаны на задокументированных доказательствах, утвержденных государственными чиновниками и сверяемых с международными критериями.

**Результаты:**

Наблюдался высокий уровень реализации некоторых показателей: предоставление списков ингредиентов и заявления о питательных веществах и регулирование заявлений о полезности для здоровья на упаковках пищевых продуктов, прозрачность процесса разработки политики, мониторинг распространенности неинфекционных заболеваний и мониторинг факторов риска неинфекционных заболеваний. Степень реализации следующих показателей была низкой (или же показатели не были реализованы вообще): ограничения рекламы нездоровой пищи для детей, фискальная политика и политика в сфере розничной торговли продовольственными продуктами, а также защита национальных условий питания в торговых соглашениях. Согласованность заключений различных экспертов составила 0,78 (95 % доверительный интервал, ДИ: 0,76–0,79). На основании пробелов в реализации эксперты порекомендовали 34 мероприятия, уделяя первостепенное внимание семи из них.

**Вывод:**

Индекс эффективности политики здорового питания предлагает набор показателей, которые можно использовать для определения конкретных областей, где необходимы действия правительства. Ожидается, что этот индекс приведет к повышению ответственности правительств, стимулирует деятельность правительства и поддержит усилия по защите гражданского общества.

## Introduction

In the past 30 years, the global proportion of overweight and obese adults has increased from approximately 29% to 37% in men, and 30% to 38% in women.^1^ The surging global burden of obesity and diet-related noncommunicable diseases (NCDs) is mainly driven by unhealthy diets.^1^^,^^2^ Unhealthy diets, in turn, are driven by unhealthy food environments.^3^ Food environments are the collective physical, economic, policy and sociocultural surroundings, opportunities and conditions that influence people’s food choices and nutritional status.^4^ Comprehensive actions by governments and the food industry are needed to achieve World Health Organization (WHO) targets to halt the rise in obesity and diabetes and reduce NCDs by 25% by 2025.^5^The International Network for Food and Obesity/Non-communicable Diseases Research, Monitoring and Action Support (INFORMAS)^4^ was recently founded to monitor and benchmark food environments, relevant government policies and private sector actions globally. Its objective is to reduce obesity and diet-related NCDs by increasing accountability and action within governments and the food industry. Accountability for prevention of NCDs at the national level is essential to ensure progress.^6^ INFORMAS complements existing monitoring efforts by WHO, such as the *Global NCD monitoring framework*, which includes few upstream indicators on food environments.^5^^,^^6^

Currently, to our knowledge, tools to assess the extent of policy implementation on food environments by governments are lacking. *The hunger and nutrition commitment index* ranks governments on their political commitment to tackling hunger and undernutrition,^7^ but does not measure the extent of policy implementation.

INFORMAS has developed the healthy food environment policy index to assess the extent of government policy implementation on food environments in comparison with international best practice.^8^ The index comprises a policy component with seven domains on specific aspects of food environments (food composition, labelling, marketing, provision, retail, prices and trade), and an infrastructure support component with six domains (leadership, governance, funding and resources, monitoring and intelligence, platforms for interaction and health-in-all-policies). The latter are based on the WHO building blocks for health systems.^9^

The healthy food environment policy index is consistent with recommended policies for countries included in WHO’s *Global action plan for the prevention and control of noncommunicable diseases 2013–2020*.^5^ For each policy index domain, a set of good practice indicators has been developed^8^ and pilot tested.^10^ The rating process,^8^ which assesses a government’s level of implementation of policy and infrastructure support, has also been pilot tested.^10^

The aim of this study was to use the healthy food environment policy index to assess the extent of implementation of national food environment policies in New Zealand. A national expert panel assessed the extent of implementation of policies on food environments by the Government of New Zealand in comparison with international best practice. The panel identified and prioritized concrete actions to increase the healthiness of food environments and reduce obesity and diet-related NCDs.

## Methods

The study was approved by the University of Auckland Human Participants Ethics Committee (reference number 9326).

### National expert panel

The expert panel comprised a comprehensive national group of informed public health and nutrition experts, including academics, representatives of nongovernmental organizations (NGOs), Māori and Pacific NGOs and medical associations. Individuals working for the government or the food industry were excluded. The experts signed an informed consent form and declared their conflicts of interest. Participation in government advisory committees was not considered a conflict of interest. Many of the experts had previously contributed to testing the methods for the healthy food environment policy index.^10^

### Compilation of evidence

Evidence on the current extent of government implementation of actions and policies on food environments and infrastructure support, was collected between February and August 2013 for all good practice indicators within the policy index domains. The full list of good practice indicators can be found as online supplementary material (Appendix A; available at: http://ebooks.fmhs.auckland.ac.nz/informas-bfe-report-2014/ ). Searches for government documents and budget information were conducted on governmental websites, libraries, via contact with government officials and via submission of official information requests. Most assessments were concerned with the current level of implementation. However, assessment of monitoring and intelligence took a longer view, since some surveys (e.g. food consumption surveys) are carried out only every few years.

The evidence collection focused on the national government, but took into account actions at subnational levels where relevant (e.g. funding for population-based nutrition promotion and food retail actions by councils, public health units and district health boards) to avoid underestimation of the extent of policy implementation.

### Validation

To verify the accuracy and completeness of the evidence collected, it was fed back to officials in the relevant government agencies, including the Ministries of Health, Primary Industries, Education, Foreign Affairs and Trade and Social Development, the State Services Commission and the Health Promotion Agency. The evidence was then updated for use in the pilot test^10^ in November 2013 and updated again for use in the rating workshops in April–May 2014. A summary of the evidence used for each good practice indicator can be found in Appendix A.

### International benchmarks

International best practice examples were derived from the World Cancer Research Fund NOURISHING database^11^^,^^12^ or obtained from international experts. Example policies include the 10% soda and 8% junk food taxes recently implemented in Mexico, sodium targets in a range of food product categories specified by law in Argentina and South Africa and the nutrient profiling system to prevent unhealthy food products carrying health claims in Australia and New Zealand. Examples for infrastructure support include Australia’s Healthy Together Victoria, a systems-based approach to obesity prevention, and England’s National Child Measurement Programme.

### Rating workshops

All experts from the national expert panel were invited to participate in one of two whole-day rating workshops in April–May 2014. Government officials were invited as observers. During the workshops, good practice indicators and evidence on the extent of implementation were presented separately for each domain and good practice indicator. After plenary discussions, each expert independently scored the current degree of implementation towards best practice for each item on a Likert scale from one to five. Qwizdom Actionpoint software (Qwizdom, Belfast, United Kingdom of Great Britain and Northern Ireland) was used to classify each indicator as follows: 1 = less than 20% implementation; 2 = 20–40% implementation; 3 = 40–60% implementation; 4 = 60–80% implementation; 5 = 80–100% implementation; unknown (cannot rate).

During the second part of the workshop, the distribution of ratings was presented for each of the good practice indicators, and through plenary discussions, concrete actions for implementation were identified by the experts. These actions were considered to have the greatest potential to improve the healthiness of food environments and reduce obesity and diet-related NCDs in New Zealand.

### Prioritization of proposed actions

Experts were provided with the rating results and the proposed actions in spreadsheet format. Policy and infrastructure support actions were prioritized separately, in the following steps. First, each expert was allocated a fixed number of points: 75 points for policy actions and 95 points for infrastructure support actions. Individual participants scored the importance of each action separately, taking into account relative need, impact, equity and other positive and negative effects. Second, the likely achievability of the action was scored, taking into account the relative feasibility, acceptability, affordability and efficiency of the action. Finally, participants were given the opportunity to apply separate weightings to the importance and achievability criteria.

### Data analysis

#### Ratings

The average rating for each indicator was used to categorize the level of implementation against international best practice as high (more than 75% implemented), medium (51–75% implemented), low (26–50% implemented) or very little, if any (less than 25% implemented). The Gwet AC2 interrater reliability coefficient and its variance were determined using AgreeStat software (Agreestat 2013.1, Advanced Analytics, Gaithersburg, United States of America). Interrater reliability was estimated as the percentage agreement between experts, with quadratic weights. For estimation of the variance, the sample of subjects to rate was set at 100% since all indicators of the healthy food environment policy index were included for rating, while the sample of raters was set at 50%, and the finite population correction was applied.^13^

#### Prioritization

The weights that the experts allocated to importance and achievability were applied to their individual scores and the scores for importance and achievability were then summed for each proposed action. Actions were ranked from higher to lower priority.

## Results

In total 52 public health experts, academics and NGO representatives (about 50% of those invited) participated in a rating workshop. Of those, 22 experts were academics, 21 were NGO representatives and nine were representatives of other organizations. Of these, one expert was of Indian ethnicity, two were European, 13 were of Māori, Pacific or mixed ethnicity and 36 were New Zealand-European ethnicity. In total 58 experts from the National Expert Panel participated in the prioritization process. None of the invited experts were excluded because of conflicts of interest.

### Ratings of the extent of implementation

**Fig. 1:**
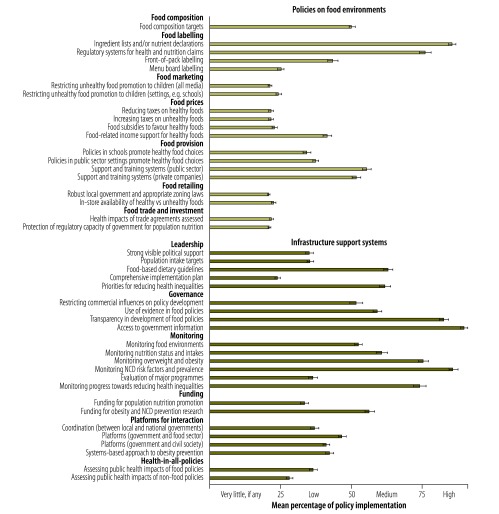
Implementation of policies on food environments and infrastructure support systems by the Government of New Zealand, compared with international best practice

The mean percentages of implementation towards best practice for policy and infrastructure support indicators are shown in Fig. 1.

NCD: noncommunicable diseases.

Note: The whiskers represent standard errors.

The experts rated the level of implementation as high for providing ingredient lists and nutrient declarations on packaged foods, regulating health claims on food products through the use of a nutrient profiling system, transparency in policy development processes, providing access to information for the public and monitoring prevalence of NCDs and NCD risk factors, including body mass index. The experts rated the extent of implementation as very little, if any for restrictions on unhealthy food marketing to children, fiscal and food retail policies and protection of national food environments within trade and investment agreements.

Interrater reliability was 0.78 (95% confidence interval, CI: 0.76–0.79), and was similar among public health academics (0.81, 95% CI: 0.79–0.83) and representatives of NGOs and medical associations (0.76, 95% CI: 0.74–0.79).

### Priority actions

Thirty-four concrete actions (Table 1 and Table 2) were identified by the experts as having the potential, in concert with other actions, to improve the healthiness of food environments and to reduce obesity and diet-related NCDs in New Zealand. Four infrastructure support actions and six policy actions were identified as the top priorities for implementation by the Government of New Zealand (Table 1 and Table 2). These were summarized as seven recommendations: (i) implementing a comprehensive national action plan for obesity and NCD prevention; (ii) setting priorities in statements of intent and setting targets for reducing childhood and adolescent obesity, reducing salt, sugar and saturated fat intake and food composition (salt and saturated fat in key food groups); (iii) increasing the funding for population nutrition promotion; doubling it to at least 70 million New Zealand dollars (NZ$) per year; (iv) reducing the marketing of unhealthy foods to children and adolescents through broadcast and non-broadcast media and in settings such as schools; (v) ensuring that foods provided in, or sold by, schools and early childhood education services meet dietary guidelines; (vi) implementing the Health Star Rating food labelling system (using stars to allow consumers to compare the nutritional profile of packaged foods); and (vii) introducing an excise tax of at least 20% on sugar-sweetened beverages.

**Table 1:** Proposed policy actions on food environments for the Government of New Zealand, prioritized by experts

Proposed policy actions	Score (% of total points allocated)^a^	Rank
**To improve food composition, the government:**	372 (8.6)	1
**– sets sodium targets for the food groups that are major contributors to sodium intake, based on international best practice targets;**		
**– establishes a food standard to minimize the unhealthy fatty acid content of commercial deep frying fats; and**		
**– examines other opportunities to reduce the amount of salt, sugar and saturated fat in foods and beverages.**		
To improve food labelling (nutrient disclosure), the government:	260 (6.0)	10
– requires trans fats to be added in the nutrition information panel where they exceed a particular level; and		
– examines the potential for including ‘added sugars’ in the nutrition information panel.		
To improve food labelling (preventing misleading claims), the government investigates the application of the nutrient profiling scoring criterion^14^ to restrict the use of nutrient content claims on packaged unhealthy foods (especially irrelevant claims such as no cholesterol claims on plant-based foods).	278 (6.4)	8
**To improve food labelling (consumer-friendly nutrition quality labels), the government endorses the Health Star Rating system for implementation from 2014 on a voluntary basis with provision to move to regulations if there is not wide coverage within two years.**	329 (7.6)	5
To improve food labelling (energy disclosure), the government requires all quick service chain restaurants to display kJ labelling (per serving as sold) on their menu boards.	242 (5.6)	12
**To reduce unhealthy food promotion to children, the government introduces regulations to restrict the marketing of unhealthy foods, as defined by the nutrient profiling scoring criterion to children and adolescents (e.g. younger than 16 years) through:**	364 (8.4)	2
**– broadcast media, with initial priorities for restriction of advertising through television; and**		
**– non-broadcast media, with initial priorities for restriction of advertising through sports sponsorship, food packaging and point-of-sale advertising.**		
**To reduce unhealthy food promotion to children, the government implements policies to ensure that schools and early childhood education and care services, are free of commercial promotion of unhealthy foods, as defined by the MoH food and beverage classification system.**	341 (7.8)	3
**To discourage the consumption of unhealthy foods and beverages, the government:**	320 (7.4)	6
**– introduces a significant (at least 20%) excise tax on sugar-sweetened beverages; and**		
**– explores how the tax revenue could be applied to create healthy food environments and promote healthy diets.**		
To ensure that taxpayer-funded food for children is healthy, the government requires all programs involving subsidised or supplied food for children (e.g. school breakfast programs) to meet the food and nutrition guidelines as outlined in the food and beverage classification system.	270 (6.2)	9
**To ensure that children’s settings provide healthy food, the government enacts policies that ensure schools and early childhood education and care services provide or sell foods which meet the food and nutrition guidelines as outlined in the food and beverage classification system.**	330 (7.6)	4
To show national leadership, the government develops and implements healthy food service policies throughout the public health sector (e.g. MoH, hospitals, DHBs, public health units).	284 (6.5)	7
To stimulate the uptake of healthy food service policies and actions, the government provides support and training systems for children’s settings, government sector and private sector workplaces (particularly small to medium businesses).	236 (5.4)	14
To support local communities achieve healthy food environments for children, the government reviews the adequacy of the current local government legislation with a view to strengthening local governments’ authority to create healthy food environments for children (e.g. ensuring ‘green food zones’ around schools to minimize unhealthy food outlets and advertising.	254 (5.9)	11
To protect the health of New Zealanders, the government includes formal and explicit population nutrition and health risk assessments as part of their national interest analysis on trade and investment agreements.	228 (5.3)	15
To avoid exposure to being sued by transnational corporations, the government ensures that specific and explicit provisions are included in trade and investment agreements allowing the Government of New Zealand to preserve its regulatory capacity to protect and promote public health.	237 (5.5)	13

DHB: District Health Board; kJ: kilo Joule; MoH: Ministry of Health.

^a^ The total points available per proposed policy action were 4345. Two out of 58 experts allocated less than 75 points. Scores cannot be compared to the scores of the infrastructure support actions which were allocated a higher number of points by the experts. Priority actions are listed in bold.

**Table 2:** Proposed infrastructure support actions for the Government of New Zealand prioritized by experts

Proposed infrastructure support actions	Score (% of total points allocated)^a^	Rank
**To demonstrate a national commitment, the government prioritizes improving nutrition and reducing childhood obesity by:**	390 (7.2)	1
**including clear support for these priorities in the government statements of intent (especially for the MoH);**		
**– setting a target to reduce the prevalence of childhood and adolescent obesity (for example, by 5% over the next six years).**		
**To demonstrate commitment and to measure progress, the government specifies clear targets for the reduction of salt, sugar and saturated fat intake of the population based on WHO recommendations and the global NCD action plan (e.g. salt intake 5 g/day, saturated fat intake less than 10% of energy, and free sugar less than 10% of energy).**	317 (5.9)	4
To ensure the consistency of policies and messages on healthy diets, the government actively implements its food-based dietary guidelines including translating and promoting them to the public and to professional groups, industry groups and relevant settings.	282 (5.2)	10
**To convert its commitments to WHO’s Global Action Plan to reduce NCDs in the New Zealand context, the government develops, funds and implements a comprehensive national action plan to prevent NCDs.**	324 (6.0)	3
To articulate the high priority to reduce health inequalities, the government embeds explicit objectives to reduce health inequalities throughout the comprehensive plan.	302 (5.6)	5
To minimize direct conflicts between commercial interests and the interests of public health nutrition, the government strengthens its conflict of interest procedures to ensure that food industry representatives with direct conflicts are not included in setting food-related policy objectives and principles (this does not apply to their participation in policy implementation).	298 (5.5)	6
To track progress towards healthier food environments and to inform action, the government strengthens its monitoring of food environments by regular:	292 (5.4)	7
– monitoring of marketing unhealthy foods to children through broadcast and non-broadcast media; and		
– monitoring the nutritional quality of foods provided and sold in schools and early childhood education and care services.		
To track progress towards healthier diets and to inform action, the government ensures that there are comprehensive regular (e.g. five yearly) food consumption surveys for adults and children, so that food and nutrient intakes and nutritional status can be assessed against nutritional and food-based guidelines and targets.	278 (5.1)	12
To track progress and to inform action at a local level, the government institutes a system to deliver regular fine-grained estimates of overweight and obesity prevalence (especially for children and adolescents) at community levels for use by local communities.	228 (4.2)	18
To track progress on NCDs and their risk factors, the government continues to invest in cardiovascular disease and diabetes risk assessments and investigates the inclusion of height and weight measurements and the use of the data for population monitoring.	280 (5.2)	11
To ensure effectiveness and the efficient use of resources, the government includes robust programme evaluation in any major investment in improving population nutrition with approximately 10% of programme costs allocated for evaluation including outcome measures.	288 (5.3)	8
To track progress and inform action on the underlying drivers of poor health and health inequalities, the government funds regular monitoring reports on the underlying societal and economic determinants of health and the related progress on the reduction of health inequalities.	288 (5.3)	9
**To ensure that sufficient resources are available to improve population nutrition, the government funding for population nutrition promotion is increased to at least NZ$ 70 million per year (equivalent to about 10% of the health-care costs of overweight/obesity and on a par with previous investments in prevention).**	339 (6.3)	2
To align research strategies with improving the healthiness of diets, the government ensures that the Science Challenges on Healthier Lives, Ageing Well, and A Better Start^b^ have a strong focus on research to improve nutrition.	232 (4.3)	17
To facilitate whole-of-government approaches to improving population nutrition and obesity, the government establishes cross-government mechanisms (national to local and between ministries) to coordinate food-related prevention policies (e.g. through the introduction of a new public sector challenge).	264 (4.9)	14
To maximize the input and value from civil society, the government ensures there are formal platforms including a nutrition advisory committee and other mechanisms for civil society organizations to be involved proactively in food policy and programme development, implementation and evaluation.	264 (4.9)	15
To maximize the impact of community-based programmes for obesity prevention, the government implements the Healthy Families New Zealand programme to at least the level of comprehensiveness, coverage and depth as the Healthy Together Victoria programme in Australia.	273 (5.0)	13
To ensure that food policies are compatible with the objectives of improving population nutrition and reducing obesity and diet-related NCDs, the Ministry for Primary Industries and the Ministry of Business, Innovation, and Employment assess the wider health impact of food policies (not only from a safety point of view) on long-term population health.	246 (4.6)	16
To ensure that government policies in general are compatible with the objectives of improving health, the government establishes a health impact assessment capacity, including funding for such capacity at the national and local level.	227 (4.2)	19

MoH: Ministry of Health; NCD: noncommunicable diseases; NZ$: New Zealand dollars; WHO: World Health Organization.

^a^ The total points available per proposed policy action were 5412. One out of 58 experts did not prioritize the infrastructure support actions and one out of 58 experts allocated 92 instead of 95 points. Scores cannot be compared to the scores of the policy actions which were allocated a lower number of points by the experts.

^b^ The National Science Challenges aim to align and focus New Zealand's research on large and complex issues by drawing scientists together from different institutions and across disciplines to achieve a common goal through collaboration.

Priority actions are listed in bold.

## Discussion

The Government of New Zealand’s level of implementation meets international best practice for some of the indicators, such as food labelling, and monitoring prevalence of NCDs and their risk factors. However, the level of implementation for over half of the good practice indicators was rated as very little, if any, or low.

According to the experts, a comprehensive national action plan, including targets to reduce childhood obesity, diet-related NCDs and population intakes of nutrients of concern is needed. Food reformulation targets (e.g. sodium in foods and saturated fat in commercial frying fats) and effective policies to improve population diets by increasing the availability, accessibility and affordability of healthy foods are also fundamental. The healthy eating – healthy action strategic framework and its associated implementation plan were abandoned in 2010.^15^

Government funding for population nutrition promotion in 2012–2013 was NZ$ 29 million, which is less than 5% of the total health-care costs of treating obesity in New Zealand.^16^ New funding of NZ$ 40 million over four years, recently announced for *Healthy Families New Zealand*,^17^ a systems-based approach to obesity prevention, is a good first step, but the experts recommended increasing population nutrition promotion funding to at least NZ$ 70 million a year, corresponding to previous prevention efforts.

The experts included the restriction of unhealthy food marketing to children through all media as one of the top priorities for improving the healthiness of food environments. Government regulation of food marketing to children is essential, since previous research in New Zealand, as well as internationally, has shown that self-regulation by the advertising industry has not reduced children’s levels of exposure to unhealthy food marketing.^18^^,^^19^ Some countries (e.g. Brazil, Chile, Peru and South Africa) recently approved legislation to restrict unhealthy food marketing to children through all forms of media,^11^^,^^12^^,^^20^ but this is yet to be implemented.

The New Zealand HeartSAFE initiative^21^ and the Chip Group initiative,^22^ which are partly funded by the ministry of health, have contributed to some reductions in the sodium (salt) content of certain food products and levels of saturated fats in commercial frying fats. The experts recommended that the government set clear reformulation targets for sodium in food products and saturated fats in commercial frying fats. This recommendation has some international precedents. Argentina and South Africa have specified maximum levels of sodium in a range of food categories by law.^11^ The United Kingdom’s salt reduction programme is a comprehensive voluntary approach, underpinned by the threat of legislation and regulation of sodium levels in food groups by the UK Department of Health. Since its introduction in 2003–2004, the sodium content in many processed foods has decreased and there has been a 15% reduction in 24-hour urinary sodium levels over seven years (reducing from 9.5 to 8.1 g per day).^23^

To better inform consumers about the healthiness of packaged foods and to further stimulate food industry reformulation, the experts recommended the implementation of the Health Star Rating system, recently introduced in Australia, as a priority. Since the finalization of this study, the Government of New Zealand has decided to adopt this system. If there is insufficient uptake by industry, the system should become mandatory. Other countries with evidence-based front-of-pack labelling systems include Ecuador (mandatory multiple traffic lights) and the United Kingdom (voluntary multiple traffic lights).^11^^,^^12^

The expert panel also recommended introducing policies to ensure that schools only provide and sell foods that meet the ministry of health food and nutrition guidelines. In Australia, five states or territories have implemented mandatory standards in schools based on either the national voluntary guidelines or nutrient and food criteria defined by the state. Unhealthy foods are either completely banned in schools or heavily restricted.^11^

Another top priority was discouraging consumption of sugar-sweetened beverages by increasing the price through an excise tax. The revenue from the tax can be used for improving population nutrition. It was recently estimated that a 20% tax on such beverages in New Zealand would avert or postpone 67 deaths (0.2% of all yearly deaths in New Zealand) from diet-related NCDs,^24^ and result in NZ$ 40 million per year in revenues.^24^ Several countries (e.g. Hungary, Mexico and Tonga) have introduced taxes on sugar-sweetened beverages and some of them use the revenue for improving population health (e.g. Hungary and Mexico).^11^^,^^12^

The healthy food environment policy index provides a useful set of indicators focusing on where government actions are needed most. The policy index method has several strengths, including the engagement of a wide group of national public health experts, the use of comprehensive evidence on the extent of implementation of food policies to support the ratings, validated by government officials, and the use of international benchmarks to rate against.

Currently there is limited evidence on the impact of several best practice examples of food policies implemented internationally. It is likely that this will improve over time and through participation of a wide range of countries in the healthy food environment policy index process. It will also be important to assess the impact of the policy index on government policies and actions through structured interviews with policy-makers and through updating the evidence on the extent of policy implementation over time.

Assessing and benchmarking the extent of implementation of government policies will increase accountability of governments for their actions on food environments. Countries of varying size and income are encouraged to apply the healthy food environment policy index to stimulate government action and support civil society advocacy efforts.
